# Pyoderma Gangrenosum as a Presenting Feature of Takayasu Arteritis

**DOI:** 10.7759/cureus.36817

**Published:** 2023-03-28

**Authors:** Wajeeha Batool, Sulhera Khan, Bareerah Khan, Marium Khan, Zeeshan Ali

**Affiliations:** 1 Department of Internal Medicine, Jinnah Postgraduate Medical Centre, Karachi, PAK; 2 Department of Internal Medicine, Dow University of Health Sciences, Civil Hospital, Karachi, PAK

**Keywords:** necrotic ulcers, skin manifestations of takayasu arteritis, american college of rheumatology, vasculitis, takayasu arteritis, pyoderma gangrenosum

## Abstract

Takayasu arteritis (TA) is a large vessel vasculitis that involves the aorta and its major branches. The disease has a female preponderance, and it presents with a wide variety of symptoms including skin manifestations, mainly ulcerative nodules, pyoderma gangrenosum, and erythema nodosum-like lesions. We report a case of a 50-year-old female who presented to the outpatient department with multiple ulcerative lesions over both upper extremities and chest. On physical examination, the patient had pulseless upper limbs. Laboratory investigations revealed positive antinuclear antibodies (ANA) and raised inflammatory markers. CT angiography of the aorta showed thickened aortic arch with the obliterated lumen of the left common carotid and left subclavian arteries. A biopsy of the skin lesion revealed surface ulceration and densely inflamed granulation tissue with a fibroblastic proliferation of deeper tissues. The patient had three out of six features of the American College of Rheumatology 1990 (ACR-1990) criteria for the classification of TA and was diagnosed with TA associated with pyoderma gangrenosum. The patient was managed with steroids and immunosuppressants along with gentle wound debridement with grafting of skin wounds. Since TA has varying presentations, its diagnosis is often challenging and requires a combined approach including clinical signs and symptoms, as well as laboratory and radiological workup. The disease also requires long-term follow-up due to its remitting and relapsing course.

## Introduction

Takayasu arteritis (TA) or Martorell syndrome was first identified by Mitiko Takayasu, a Japanese ophthalmologist who first recognized arteriovenous malformations in the retina in 1908 [1]. TA is a large vessel vasculitis of unknown etiology involving the aorta and its branches [2]. Initially, it was believed that TA predominantly affected females from East Asian countries such as Japan, and India; however, recent studies and case reports show that the disease is prevalent all over the world [3]. The overall prevalence of the disease is 3.3/million people worldwide [4]. Due to the low prevalence of TA and scarcity of data among the Pakistani population, the exact prevalence of the disease in Pakistan is not known. The disease has a female preponderance (9:1) with most patients diagnosed in the second or third decades of life [5].

TA is a chronic inflammatory arteritis leading to vessel thickening, fibrosis, stenosis, and thrombus formation [5]. Clinical manifestations of the disease can be classified into non-specific and specific symptoms. The non-specific symptoms include fever, malaise, and weight loss. However, specific features arise due to arterial insufficiency such as intermittent claudication, bruits, loss of pulses, and blood pressure [2]. TA also presents with skin manifestations including erythema nodosum and pyoderma gangrenosum-like ulcers. Pyoderma gangrenosum is an ulcerative cutaneous condition characterized by painful necrotic ulcers and sterile neutrophilic infiltration of the dermis. This condition is associated with various inflammatory and autoimmune diseases [3]. Our case is of significance as our patient presented with deep-seated ulcers along with signs of arterial insufficiency and was later diagnosed with TA with pyoderma gangrenosum, an uncommon but significant association.

## Case presentation

A 50-year-old female with no known comorbidities presented to the outpatient department with multiple ulcerative lesions involving both upper extremities and breasts for the last four months. The patient had sought medical attention at local clinics where she had been initially managed with intravenous antibiotics (meropenem and vancomycin) with no response. The lesions had begun as small painful shallow ulcers that had gradually enlarged and become deep. There was no history of oral and anogenital ulcers. The patient also gave a history of frequent headaches more pronounced over temporal areas, relieved temporarily with analgesics. The headache had significantly impaired her daily activities of life. She also complained of intermittent jaw claudication as well as upper and lower limb claudication relieved with rest. She had also experienced a transient loss of vision in her right eye a month ago. Upon further evaluation, the patient stated that she had been experiencing low-grade undocumented fever, reduced appetite, and malaise for the past several months. She also complained of alopecia and Raynaud’s phenomenon. The patient denied any alcohol and nicotine abuse, and urinary, respiratory, or gastrointestinal complaints.

**Figure 1:**
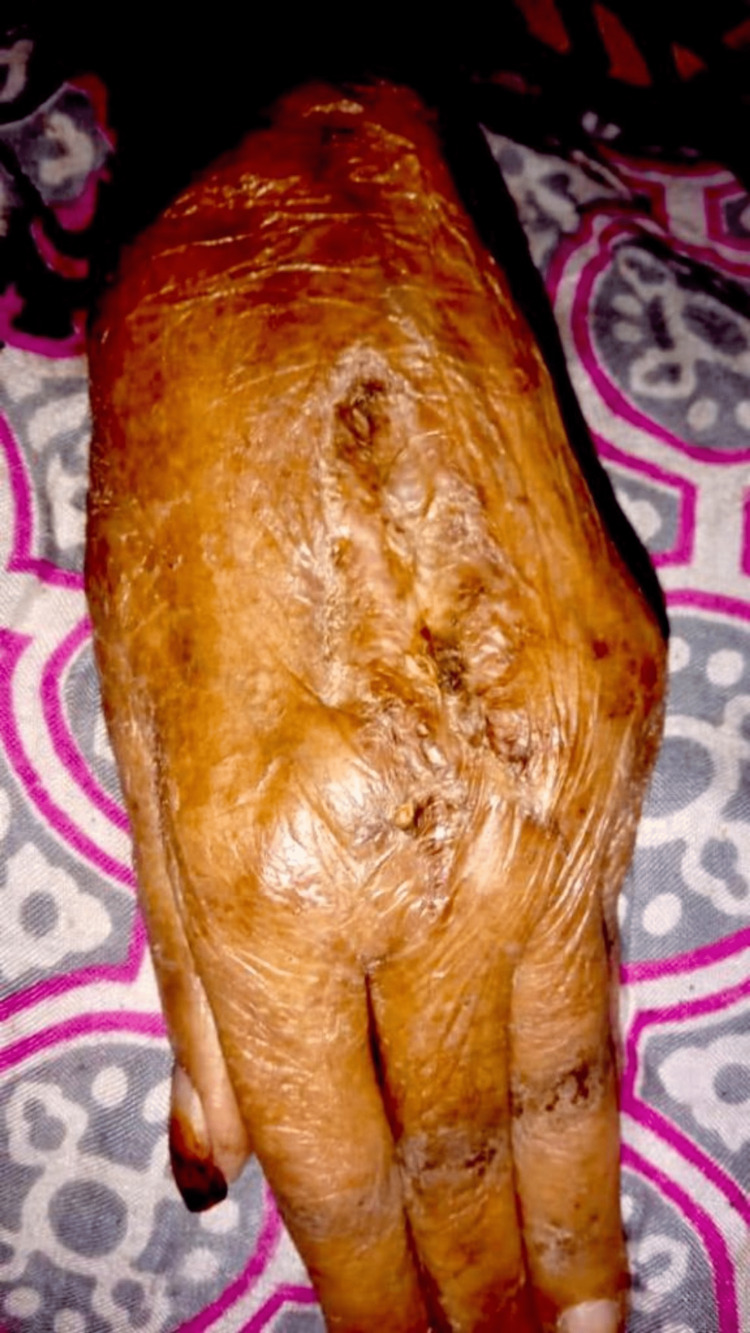
The hand of the patient showing multiple ill-defined atrophic plaques with peripheral scaling

**Figure 2:**
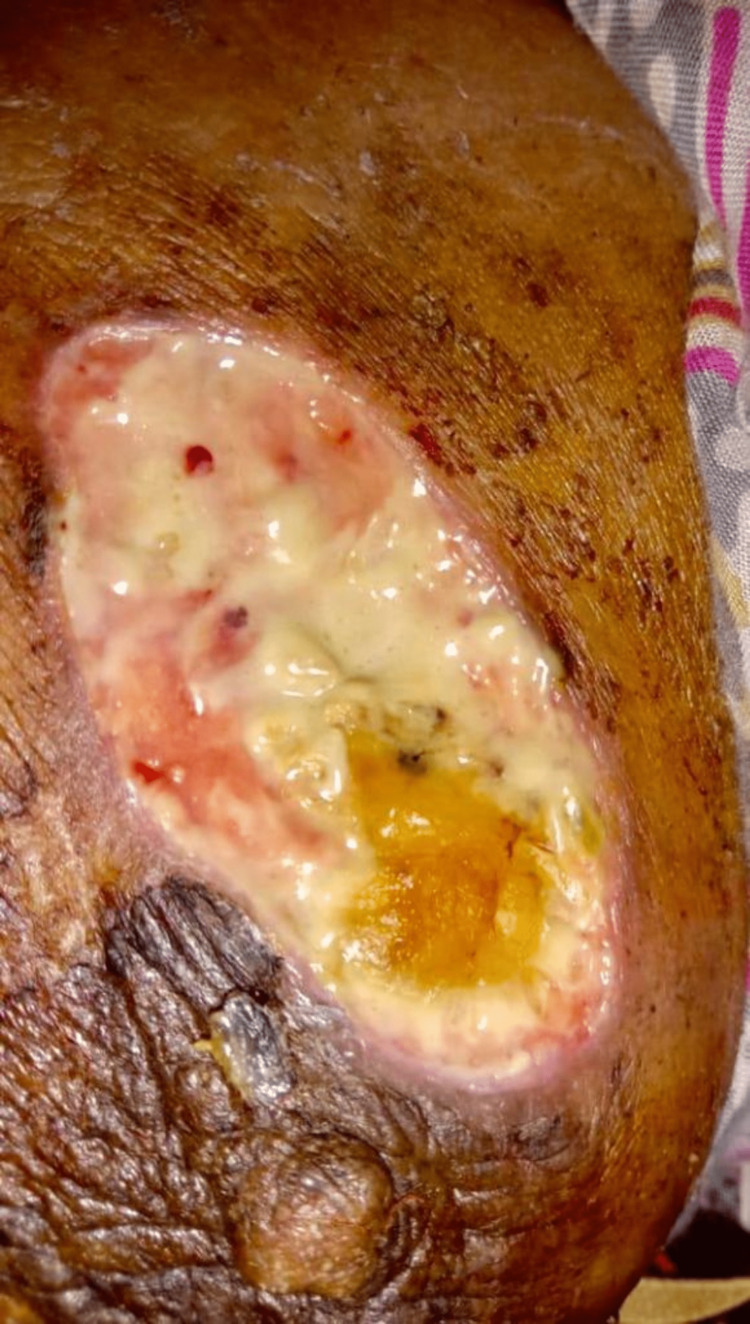
A well-defined ulcer with undermined edges and violaceous borders with active purulent discharge at the base of the ulcer present over the 12 o'clock position of the breast also involving the areola

On examination, the patient was alert and well-oriented. On examination of the vitals, the patient was pulseless in both upper limbs, while the lower limb pulses were palpable. Blood pressure was undetectable in both arms (0 mmHg) while it was 130/76 mmHg in the left lower limb and 136/79 mmHg in the right lower limb. Temporal arteries were palpable bilaterally and were non-tender. There was an audible bruit over the left carotid artery. Carotidynia was also positive, predominantly on the right side. On cardiovascular examination, the patient had a heart rate of 96 beats per minute and no additional heart sounds or murmurs were audible. Chest, abdominal, and neurologic examinations revealed no significant findings. Local examination of hands revealed multiple ill-defined atrophic plaques with peripheral scaling (Figure 1). A breast examination revealed a well-defined ulcer with undermined edges and violaceous borders with active purulent discharge at the base of the ulcer (Figure 2).

**Figure 3:**
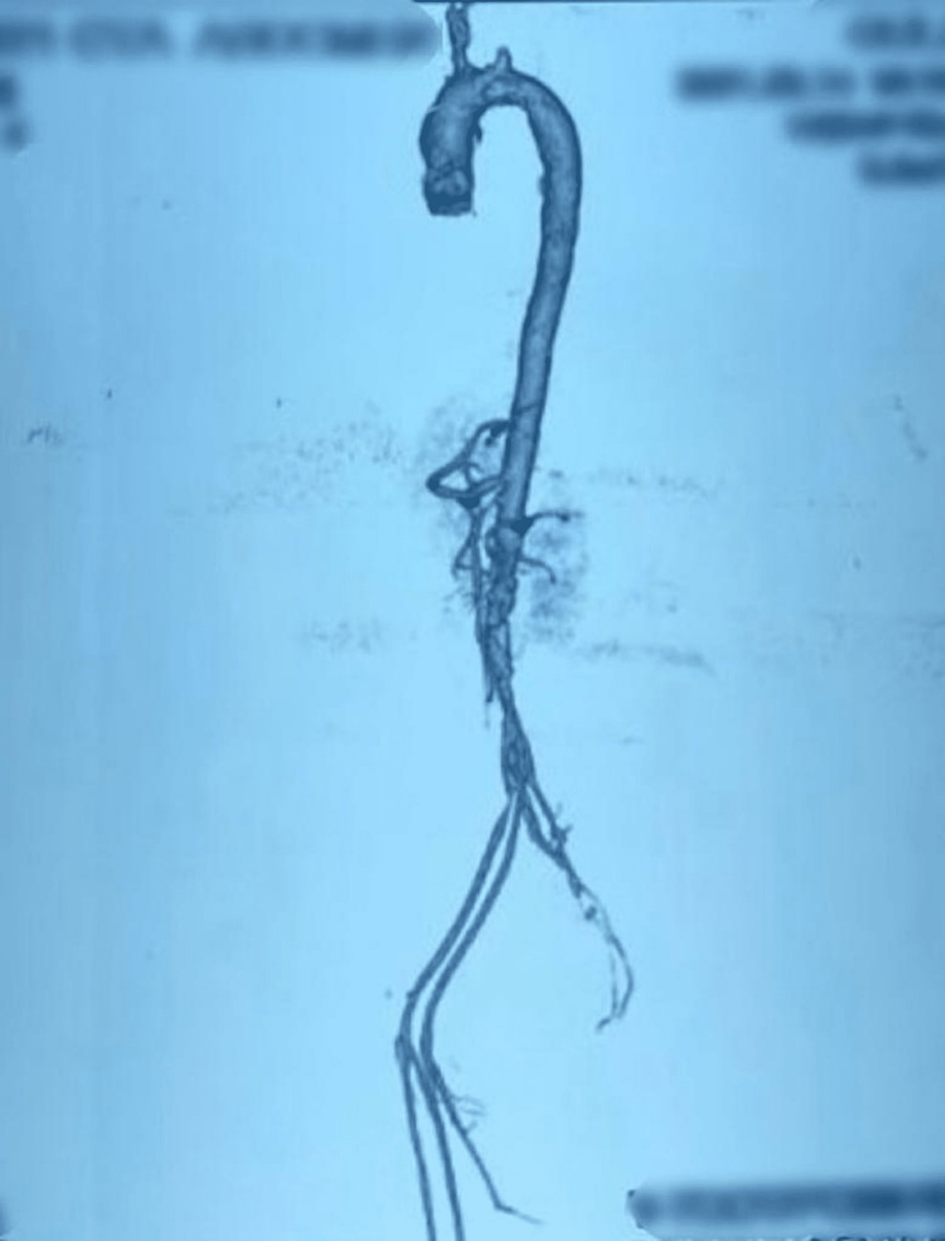
CT angiography of aorta showing thickened aortic arch and non-visualized left common carotid and left subclavian arteries CT: computed tomography

The patient was worked up for vasculitis based on her history and examination findings. Complete blood count showed mild anemia (Hb: 10.5 g/dl), and neutrophilic leukocytosis (17.3 x 10^9^/L white cells with 88% neutrophils). Inflammatory markers were significantly raised (ESR: 90 mm/1st hour, CRP: 58 mg/dl). Pus culture (taken from an ulcer on breasts) revealed growth of Pseudomonas aeruginosa sensitive to various broad-spectrum antibiotics. Antinuclear antibody (ANA) was positive with a speckled pattern and 1/160 estimated endpoint titer. However, antibodies to extractable nuclear antigens (ENA), as well as anti-neutrophilic cytoplasm (ANCA), were negative. Lipid profile (serum cholesterol, triglycerides, low-density lipoprotein levels), urinalysis, coagulation profile, and renal and liver function tests were within normal limits. All laboratory investigations are summarized in (Table 1). Doppler ultrasound of both upper extremities showed moderate thickening of subclavian vessels with reduced flow velocity while the lumen of ulnar and radial arteries was completely obliterated. Doppler imaging of both superficial temporal arteries showed moderate thickening while the vessels were normal in the lower extremities. CT angiography of the aorta (Figure 3) revealed circumferential mural thickening of the arch of the aorta. There was a complete obliteration of the lumen of the left common carotid and subclavian artery with non-opacification of the vessels. The rest of the major aortic branches appeared normal. A biopsy of skin lesions on the breast was done once the infection was resolved; it showed skin and deeper adipose tissue with surface ulceration and densely inflamed granulation tissue. There was a fibroblastic proliferation in deeper subcutaneous tissue with normal medium-caliber blood vessels. These findings were suggestive of pyoderma gangrenosum. The histopathology specimen was sent for acid-fast bacilli and GeneXpert tests for tuberculosis but the results came back negative. However, bacterial and fungal cultures could not be performed due to financial constraints. The direct immunofluorescence was positive with perivascular deposition of the immune reactants.

**Table 1:** Laboratory investigations ANA: antinuclear antibody; AMA: antimitochondrial antibody; Anti-dsDNA: anti-double-stranded DNA antibody; Anti-SS-A/B: anti-single-stranded A/B; U1-RNP: U1 small nuclear ribonucleoprotein; Anti-Scl-70: anti-topoisomerase antibody; Anti-Sm: anti-Smith antibody; ASMA: anti-smooth muscle antibody; Anti-SLA: anti-soluble liver antibody; Anti-LKM1: anti-liver-kidney-muscle antibody; IgG: immunoglobulin G; Anti-MPO(p-ANCA): anti-neutrophil cytoplasmic autoantibody; Anti-PR3 (c-ANCA): perinuclear anti-neutrophil cytoplasmic antibody; HDL: high-density lipoprotein; LDL: low-density lipoprotein

Investigations	On admission	Eighth day post-admission
Complete blood count		
Hemoglobin	10.2 g/dl	10.5 g/dl
Mean corpuscular volume	84 fl	82 fl
Hematocrit	30.6%	31.5%
Platelets	225 x 10^9^/L	314 x 10^9^/L
White blood cells	17.3 x 10^9^/L	8.9 x 10^9^/L
Neutrophils	88%	69%
Lymphocytes	10%	23.6%
Eosinophils	0.4%	0.5%
Monocytes	1.4%	6.6%
Basophils	0.2%	0.3%
C-reactive protein	58 mg/dl	12 mg/dl
Erythrocyte sedimentation rate	90 mm/1^st^ hour	42 mm/1^st^ hour
Liver function tests		
Total bilirubin	1.06 mg/dl	1.11 mg/dl
Direct bilirubin	0.48 mg/dl	0.49 mg/dl
Aspartate transaminase	26 U/L	18 U/L
Alanine transaminase	19 U/L	13 U/L
Alkaline phosphatase	100 U/L	125 U/L
Gamma-glutamyl transferase	16 U/L	25 U/L
Albumin	4.2 mg/dl	4.1 mg/dl
Coagulation profile		
Prothrombin time	11 seconds	10 seconds
International normalized ratio	0.9	0.83
Activated partial thromboplastin time	20 seconds	22 seconds
Basic metabolic panel		
Sodium	138 mEq/L	139 mEq/L
Potassium	4.2 mEq/L	4.4 mEq/L
Chloride	88 mEq/L	90 mEq/L
Creatinine	0.7 mg/dl	0.9 mg/dl
Blood urea nitrogen	8 mg/dl	12 mg/dl
Glucose	96 mg/dl	106 mg/dl
Autoimmune profile; ANA, AMA, Anti-dsDNA, Anti-SS-A/B, Anti-U1-RNP, Anti-Scl-70, Anti-Sm, ASMA, Anti-SLA, Anti-LKM, Serum IgG, Anti-MPO(p-ANCA), Anti-PR3 (c-ANCA)	ANA positive (speckled) 1/160; all others negative	
Lipid profile		
Total cholesterol	146 mg/dl	
HDL cholesterol	39 mg/dl	
LDL cholesterol	76.6 mg/dl	
Serum triglycerides	115 mg/dl	

Based on the above findings, the patient was diagnosed with TA with pyoderma gangrenosum. She was managed initially with high-dose oral prednisone (1 mg/kg) for two weeks with the dose gradually tapered to 20 mg/day thereafter. Bone protection with calcium and vitamin D supplements was also given. Methotrexate (15 mg/week) was also started concomitantly along with folic acid. Low-dose aspirin (75 mg/day) was also commenced. Broad-spectrum antibiotics were given due to the superimposed infection of skin wounds. The lesions were initially washed daily with sterile saline and covered with moist dressing but later on, the patient had to go undergo gentle wound debridement with skin grafting as the lesions were not healing and causing debilitating pain. The patient was discharged upon improvement with advice for close follow-up after detailed counseling was done regarding the fluctuating course of the disease.

## Discussion

TA is a granulomatous inflammatory vasculitis that involves medium to large arteries causing transmural thickening of the vessel wall as well as degeneration of elastic tissue. This leads to occlusion and eventually ischemic changes along with the formation of alternative areas of aneurysms in the vessel wall [1]. The exact etiology of the disease is unknown, but it is thought that autoimmunity involving cell-mediated pathways might be responsible [1,6]. It has been seen that the aorta and subclavian and carotid arteries are most commonly affected by the disease process [7]. In 80-90% of the cases, the disease affects young women between 10-40 years of age. Clinical presentation ranges from asymptomatic disease or non-specific constitutional symptoms in the early stage, causing missed or delayed diagnosis. Once the disease progresses, classical signs of arterial insufficiency develop, including neurologic complications (in case of carotid artery involvement), upper limb claudication (subclavian artery stenosis), hypertension (renal artery involvement), or ischemic heart disease if coronary arteries get involved. Although rarely seen, TA may also have cutaneous manifestations that mainly include pyoderma gangrenosum, erythema nodosum, and erythema induratum [8]. In our case, the patient presented with well-defined ulcers on the breast with undermined edges, leading to a diagnosis of pyoderma gangrenosum. On further clinical evaluation, we discovered that the patient had a long-standing history of non-specific constitutional symptoms for which she had not sought medical attention. Zhang and Jiao have also reported two cases of young women in China who presented with skin lesions that were diagnosed as pyoderma gangrenosum associated with TA [9]. Werfel et al. in their case report have discussed a young Turkish woman with nodular lesions of lower extremities that were previously diagnosed as erythema nodosum [8]. However, a deep excision biopsy from a lesion showed granulomatous vasculitic changes that are atypical of erythema nodosum. The patient also had stenosis of subclavian and vertebral arteries evident on angiography and was subsequently diagnosed as a case of TA with erythema nodosum-like skin manifestations.

TA is diagnosed by a combination of clinical signs and symptoms, inflammatory markers, and radiologic imaging. The American College of Rheumatology 1990 (ACR-1990) criteria for the classification of TA provide the diagnostic guidelines for TA. This includes six features of which three must be present for a diagnosis: onset at age less than or equal to 40 years; lower or upper limb claudication; low brachial pulse; a difference of greater than 10 mmHg in systolic blood pressure between the two arms; subclavian artery or aortic bruit; and evidence of narrowing of the entire aorta, primary branches or branches to the upper or lower limbs on arteriography [10]. In our patient, three features were present, thereby fulfilling ACR criteria for TA. The mainstay of management is the use of steroids. However, to avoid steroid-induced side effects, steroid-sparing immunosuppressive agents are also used. Complications associated with TA should be managed properly. In our patient, the lesions were managed initially with a sterile, moist dressing, but due to extensive necrotic tissue with superimposed recurrent infections, the patient underwent gentle wound debridement with skin grafting. The patient’s clinical condition improved and she was discharged on steroids and methotrexate with regular follow-ups on an outpatient basis.

## Conclusions

TA presents with a wide variety of symptoms, making its diagnosis challenging. Although cutaneous lesions are not commonly seen in TA, patients with skin lesions of unknown etiology should be investigated for TA. Since the disease has a relapsing and remitting course, regular follow-up is required with an assessment of physical signs and symptoms, acute phase reactants, and radiological imaging.
